# Valedictory

**Published:** 1889-12

**Authors:** T. S. Powell, R. C. Word


### EDITORIALS AND MISCELLANEOUS.

### VALEDICTORY.

The undersigned, heretofore the Editors and Proprietors of THE SOUTHERN MEDICAL RECORD, have now to announce to their numerous readers and friends that they have sold their interest in the RECORD to Dr. D. H. HOWELL of Atlanta, Ga., who is now the proprietor, and will hereafter publish the Journal, commencing with the January number, 1889.

All amounts due the Journal up to the close of the present year, or to the 1st day of January, 1890, are due to Dr. Word, the present business manager.

All unexpired subscriptions and contracts will be carried out by the new proprietor, and all dues accruing from and after the 1st day of January, 1890, will belong to Dr. Howell, and he is authorized to collect the same.

Our readers will, therefore, please remember to remit all amounts due or to become due on the 31st of Dec., 1889, to Dr. R. C. Word, who will give attention to closing up the old business.

We take leave of our old friends and patrons with a feeling of regret and sadness, of which we have no words adequate to give expression.

Their names, many of them upon our list for a period of twenty years, are to our minds as friends true and beloved, and will ever be cherished in our memories and our hearts!

To our exchanges, too, we feel a warm degree of kindness, and express our high appreciation of the aid derived from them, and our warmest thanks for courtesies extended. They are requested to continue to exchange with the new proprietor.

The step thus taken had become a necessity to each of us by reason of advancing years, and of constantly increasing duties too onerous to be continued. It were better for us, our readers, and for the interest of the RECORD that new, young, and more active hands assume the arduous duties which the progress of the times, and the accumulating details of the Journal business demand.

This is made the more necessary by our connection with the Southern Medical College, which has assumed proportions so large as will hereafter demand much time and labor at our hands. While all this is true we would not be understood as wholly done with the pen, for we hope to find time for an occasional contribution to The Record, as we feel it the duty of every true friend of the Profession to do something for the advancement of Medical Literature, and the progress of Medical Science in his section.

We bespeak for THE RECORD the continued patronage and support of all of its old friends, and of the Profession at large.

We trust that all our readers will continue to take the Journal, and that they will give to Dr. Howell their cordial cooperation and support.

Surely our home journals, of which the RECORD stands among the first, should be sustained by the physicians in this section.

So mote it be—adieu!

T. S. POWELL, M. D.
R. C. WORD, M. D.

